# A review of the biologic and pharmacologic role of docosapentaenoic acid n-3

**DOI:** 10.12688/f1000research.2-256.v2

**Published:** 2014-08-19

**Authors:** Puya G Yazdi

**Affiliations:** 1 UC Irvine Diabetes Center, University of California at Irvine, Irvine, CA, 92697, USA; 2 Sue and Bill Gross Stem Cell Research Center, University of California at Irvine, Irvine, CA, 92697, USA; 3 Department of Medicine, University of California at Irvine, Irvine, CA, 92697, USA

## Abstract

Fish oil contains a complex mixture of omega-3 fatty acids, of which eicosapentaenoic acid (EPA), docosapentaenoic acid (DPA), and docosahexaenoic acid (DHA) are the three predominant forms. There has been a plethora of previous research on the effects and associations of fish oil supplementation with various clinical manifestations. While the majority of this work was focused on EPA and DHA as the active compounds, emerging research has begun to elucidate the specific role that DPA plays in these physiological processes and its differences with the other omega-3 fatty acids. The purpose of this review is to focus on the new studies undertaken with DPA. This review summarizes the biochemical mechanisms involved in the biosynthesis and metabolism of DPA before focusing on its effects in cardiovascular disease, immune function, and psychiatric and cognitive health. The limited studies point toward a positive role that DPA supplementation can play in these processes and that is separate and distinct from traditional supplementation with DHA and EPA.

## Introduction

As a result of anecdotal reports of their low incidence of coronary heart disease, Bang and Dyerburg began to study the Greenland Eskimo (Inuits) population in the late 1960s. Their pioneering findings confirmed the anecdotal evidence; Inuits had lower incidences of myocardial infarction, better lipid profiles, reduced platelet activity, and lower incidence of immune and inflammatory diseases compared with western controls ^1– 3^. These findings were attributed to the Inuit diet, and specifically to the large quantities of seal and whale meat consumed by the Inuits. Eventually it was deduced that marine n-3 fatty acids found in the seal and whale meat was the main protective agent against cardiovascular heart disease ^4^. These findings, along with separate research that demonstrated mammalian brain grey matter was also rich in n-3 fatty acids, became an impetus for much scientific and clinical research into the potential health benefits of n-3 fatty acids ^5, 6^. Over the ensuing years, n-3 fatty acids have been found to hold great therapeutic promise in a myriad of conditions including, but not limited to, neural function, diabetes mellitus, cardiovascular health, cancer, lipid regulation, and as a anti-inflammatory agents ^7– 12^.

The majority of the studies conducted on n-3 fatty acids were conducted on fish oils that contain a mixture of the three n-3 fatty acids: eicosapentaenoic acid (EPA), docosapentaenoic acid (DPA) and docosahexaenoic acid, (DHA). While much attention has been focused on DHA and EPA, the literature on DPA remains brief. In recent years, research on DPA has increased. Much of the increased interest in DPA came about from two observations. First, the seal meat so readily consumed by the Inuits contains high concentrations of DPA; and second, human mother’s milk also contains high concentrations of DPA ^13^. There already exists extensive literature and reviews on the beneficial effects of EPA and DHA which the reader is encouraged to read ^14– 18^. The following review will aim to address some basic biological insights behind DPA and some of its potential beneficial effects. Additionally, for a more thorough review of the biochemical and molecular pathways related to DPA, the reader is encouraged to read another recent DPA review, cited in the biosynthesis section below. This review will focus on the clinical associations involved with DPA and it will do this by focusing on DPA specific studies in addition to studies conducted on fish oils or mixtures of omega-3 n-fatty acids that contained DPA. Studies in which the role of DPA was studied specifically will be noted as well studies that were conducted on animal models or *in vitro*.

## Biosynthesis and metabolism of essential polyunsaturated fatty acids

**Figure 1.**
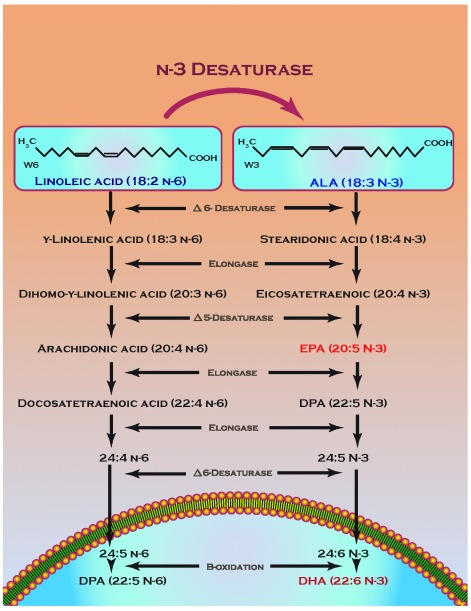
n-3 fatty acid production. Summary of the biochemical enzymes and intermediates involved in n-fatty acid production in cells. The blue underneath the lipid bilayer represents a peroxisome with steps occurring outside in the cytoplasm.

EPA, DHA, and DPA are the three major polyunsaturated fatty acids formed by a series of desaturation and elongation enzymes from Alpha-linolenic acid (ALA) ^19^. Fatty acid elongase is responsible for the direct conversion of EPA into DPA by direct chain elongation ^20– 22^. The conversion of DPA into DHA is more circuitous involving chain elongation followed by desaturation in the cytoplasm before being moved to the peroxisome to be chain shortened by β-oxidation to form DHA ^23, 24^. It is worth noting that in living cells this process can go in reverse yielding EPA, a process that most likely involves peroxisomal acyl-coA and β-oxidation ^25^. For a more thorough review of the biochemistry behind DPA the reader is encouraged to read the previously aforementioned DPA review ^25^. Figure 1 summarizes the major pathways involved in n-3 fatty acid production.

## Potential clinical effects of DPA

### Cancer

Numerous studies have reported possible anti-cancer effects of n-3 fatty acids, particularly for breast, colon, and prostate cancer ^26– 28^. Omega-3 fatty acids reduced prostate tumor growth, slowed pathological progression, and increased survival in mice ^29^. Among n-3 fatty acids, high levels of DHA, which is the most abundant n-3 polyunsaturated fat in erythrocyte membranes, were associated with a reduced risk of breast cancer ^30^. Additionally, a 2007 systematic review of n-3 fatty acids and cachexia found evidence that oral n-3 fatty acid supplementation was beneficial as adjuvant cancer therapy by improving appetite, weight, quality of life, and retaining muscle mass ^31, 32^. Additionally, a recent study looking at the anti-tumorigenic effects of n-3 fatty acids on colorectal cancer found anti-proliferative and pro-apoptotic effects for all 3 fatty acids, with DPA demonstrating the strongest effects in both *in vitro* and *in vivo* models of colorectal cancer ^33^.

### Cardiovascular disease

Not surprisingly, some of the main initial work was done on the effects of n-3 fatty acid in heart disease and specifically myocardial infarction. As early as three months and onwards, treatment with 1gram per day of n-3 fatty acids resulted in a statistically significant reduction of the occurrence of death, sudden cardiac death, and cardiovascular death ^34, 35^. It is worth noting that these trials were done on a commercial preparation of n-3 fatty acids that contained very low concentrations of DPA and hence additional work needs to be done to determine what role if any DPA can play in conferring this cardiovascular protection. Furthermore, it has also been demonstrated that n-3 fatty acids have a statistically significant antihypertensive effect by lowering systolic blood pressure by 3.5–5.5 mmHg ^36^. For a more thorough review of all the potential benefits of n-3 fatty acids in general on the cardiovascular system, the reader is advised to read a recent review ^18^.

While much of this previous work has shown that omega-3 fatty acids can possibly play a protective role in maintaining a healthy cardiovascular system, recent studies have demonstrated a positive association between DPA itself and cardiovascular disease prevention in humans ^37– 39^. A prospective population study of 1871 subjects in Eastern Finland demonstrated that subjects with plasma blood concentrations of DPA plus DHA in the 20 ^th^ percentile had a decreased relative risk of acute coronary events of 44%, compared to those subjects in bottom 20 ^th^ percentile. The decreased relative risk rose to 67% so long as the top 20 ^th^ percentile group had mercury levels below or equal to 2 micrograms/g compared to those in the bottom 20 ^th^ percentile who had mercury levels above 2 micrograms/g ^38^. This study was followed up by a Japanese study, published five years later that further corroborated these findings by showing a significant association between DPA supplementation and reduced cardiovascular disease ^37^. A nested-case control study of 6438 adults with seven years of follow up also showed that DPA was associated with a reduced the odds ratio of heart disease ^40^. More importantly, this same finding was further confirmed by another nested-case control study of 32,826 subjects with 6 years of follow up that also demonstrated that this association was independent of the other n-3 fatty acids ^41^. Interestingly, a cross-sectional study of 26 subjects and 24 matched controls revealed that decreased DPA concentrations in the cell membrane of erythrocytes (3.0+/-0.19% for subjects vs. 3.9+/-0.12% for controls, P<0.001) were statistically significantly associated with increased heart disease ^42^.

Recent studies have further developed our understanding of which pathophysiologic mechanisms DPA supplementation can potentially target in cardiovascular disease. First, a prospective cohort study which directly measured circulating levels of n-3 fatty acids in the blood of 2735 US adults without existing heart disease who were enrolled in the Cardiovascular Health Study from 1992 to 2006 found that total n-3 fatty concentration was associated with lower incidence of congestive heart failure with DPA, conferring as much as a 40% reduction ^43^. A second study looked at the impact of n-3 fatty acid on coronary plaque instability by use of conventional and integrated backscatter intravascular ultrasound approaches ^44^. Their results demonstrated that low serum concentrations of DPA were significantly associated with lipid-rich plaques, suggesting that decreased DPA levels can contribute to increased incidence of plaque formation leading to acute coronary syndrome and myocardial infarction ^44^. Besides heart disease, stroke prevention and arterial blockage also comprise a significant part of cardiovascular health. Plasma levels of DPA are inversely related to blocked arteries and DPA the only n-3 fatty acid that has been shown to significantly reduce blocked arteries regardless of lifetime smoking ^45^. A cross-sectional study with carotid ultrasonography revealed that carotid artery wall thickness was inversely associated with DPA intake ^46^.

Additionally, *in vitro* work has started to isolate the biochemical mechanisms by which DPA could help combat cardiovascular disease. For instance, platelet aggregation is an early event in the development of thrombosis and is initiated by thromboxane A2 ^47^. Recently, an *in vitro* study conducted in rabbit platelets showed that EPA, DPA and DHA inhibited collagen- or arachidonic acid stimulated platelet aggregation in a dose dependent manner, with DPA being the most potent inhibitor and possibly ten times more powerful than EPA in inhibiting platelet aggregation ^48^. A further study conducted on human whole blood corroborated these earlier findings ^49^. Endothelial cell (EC) migration and proliferation are important processes in the control of the wound-healing response of blood vessels ^50^. DPA has been shown to be a potent stimulator of EC migration ^51^. Furthermore, DPA pretreatment of bovine aortic endothelial (BAE) cells inhibits their migrating activity due to vascular endothelial growth factor (VEGF) stimulation ^9^. Additionally, that same pretreatment suppresses tube formation demonstrating that DPA is a potent inhibitor of angiogenesis, a physiological mechanism that contributes to tumor growth, inflammation, and microangiopathy ^9^.

Numerous studies have demonstrated that EPA and DHA can lower triglycerides (TG) and cholesterol levels in the plasma and liver, by increasing β-oxidation activity in the mitochondria and peroxisomes in hepatic cells, and suppressing TG synthesis in the liver ^52– 54^. Similar work is now showing that DPA also possesses lipid metabolism improving effects similar to EPA and DHA in improving cholesterol and TG levels. A recent *in vivo* study demonstrated that DPA can reduce non-HDL cholesterol by 50% in hamsters ^55^. Finally, DPA has been shown to have a positive role in reducing the expression of inflammatory genes (inflammation in the walls of blood vessels is thought to play a role in the development of atherosclerotic plaques leading to cardiovascular disease) thereby improving cardiovascular health ^56^.

### Immune function

The role of n-3 fatty acid supplementation in immune function is just beginning to emerge as a new frontier in fatty acid research. In a study regarding fish oil supplementation in infants, the authors found that fish oil supplementation could lead to quicker immune maturation without a concomitant reduction in immune activation ^57^. Additionally, owing to the anti-inflammatory properties of n-3 fatty acids and the pro-inflammatory properties of n-6 fatty acids found in most western diets, many researchers currently believe that fish oil supplementation can aid many chronic inflammatory conditions by decreasing the n-6 to n-3 fatty acid ratio ^58^. As one example, patients suffering from rheumatoid arthritis report reduced pain symptoms when taking n-3 fatty acids in conjunction with NSAIDS compared to those only taking NSAIDS ^59^. Finally, enzymatically oxygenated derivatives (oxylipins) of DPA have been shown to be potent anti-inflammatory compounds in animal models ^60^. Significant work remains to be done to elucidate the possible role if any played by DPA in immune function.

### Psychiatric and neurological function

There is now emerging evidence that n-3 fatty acids can play a role in psychiatric disorders due to the observation that schizophrenic patients demonstrate reduced levels of both n-3 and n-6 fatty acids ^61^. Treating high-risk children with a dietary supplement of n-3 fatty acids demonstrated a statistically significant decrease in progression to schizophrenia ^61^. Additionally, it has also been shown that patients with schizophrenia have decreased levels of DPA in erythrocytes ^62^. Finally, a meta-analysis based on 10 clinical trials, found that n-3 fatty acids significantly improved depression in patients with both unipolar and bipolar disorder ^63^.

Neurological and cognitive decline as a result of aging or disease is now emerging as a new avenue of further research in n-3 fatty acid supplementation. First, in a rat model of Alzheimer’s disease, EPA supplementation revealed a statistically significant efficacy in countering memory impairment ^64^. This study spurred interest in studying the possible benefit of DPA supplementation in cognitive decline due to aging. Specifically, during the aging process, there is a loss of synaptic function which leads to deficits in spatial learning tasks and reduced ability of rats to sustain long term potentiation ^65^. By supplementing the normal diets of rats with DPA it was found that DPA possesses neuro-restorative effects in the hippocampus by decreasing microglial activation and oxidative stress, the two major biochemical mechanisms involved in cognitive decline due to synaptic function loss ^66^. These authors concluded that DPA supplementation might play a significant role in neuro-protection against age-related cognitive decline by attenuating the age-related decline in spatial learning and long-term potentiation.

## Conclusions

While much work has been done on the potential therapeutic benefits of n-3 fatty acids, the majority of that work has been done on EPA and DHA. Initially, the Inuit population and their diet caught the attention of the medical community because of their much lower incidence of cardiovascular disease, which was attributed to their consumption of n-3 fatty acids. What has been forgotten in the ensuing years was that their seal meat also had high concentrations of DPA in addition to the more familiar EPA and DHA counterparts. Additionally, levels of DPA in human breast milk are high, and DPA levels in adult human blood are similar to EPA ^13^. These findings are a part of a growing body of work on DPA with promising results. Additionally, this research has also begun to elucidate important differences between DPA and EPA and DHA. Specifically, DPA inhibits platelet aggregation more efficiently than EPA or DHA, DPA stimulates endothelial cell migration much more efficiently than EPA, and finally DPA is incorporated into human plasma and red blood cell lipids faster than EPA and hence may act as a reservoir of the major n-3 fatty acids in humans ^9, 51, 67^. Studies looking into DPA and its clinical associations are beginning to demonstrate that lack of DPA in diets and blood circulation may serve as an independent predictor and marker for various health conditions. The existing evidence points to DPA as showing potential as a nutritional and therapeutic supplement. While much work still needs to be addressed on the possible benefits of DPA consumption in human health and disease, the limited data available seems to indicate that DPA can have additional health benefits in conjunction with the more common n-3 fatty acids.

## References

[1] Bang HO Dyerberg J Nielson AB: Plasma Lipid and Lipoprotein Pattern in Greenlandic West-Coast Eskimos. *Lancet.* 1971;1(7710):1143–1145 10.1016/S0140-6736(71)91658-8 4102857

[2] Dyerberg J Bang HO Aagaard O: Alpha-Linolenic Acid and Eicosapentaenoic Acid. *Lancet.* 1980;1(8161):199 10.1016/S0140-6736(80)90679-0 6101648

[3] Kromann N Green A: Epidemiological-Studies in the Upernavik District, Greenland - Incidence of Some Chronic Diseases 1950–1974. *Acta Med Scand.* 1980;208(5):401–406 10.1111/j.0954-6820.1980.tb01221.x 7457208

[4] Dyerberg J Bang HO Stoffersen E: Eicosapentanoic Acid and Prevention of Thrombosis and Atherosclerosis. *Lancet.* 1978;2(8081):117–119 10.1016/S0140-6736(78)91505-2 78322

[5] Crawford MA Sinclair AJ: Nutritional influences in the evolution of mammalian brain.In: lipids, malnutrition & the developing brain. *Ciba Found Symp.* 1971;267–292 10.1002/9780470719862.ch16 4949878

[6] Crawford MA Casperd NM Sinclair AJ: The Long-Chain Metabolites of Linoleic and Linolenic Acids in Liver and Brain in Herbivores and Carnivores. *Comp Biochem Physiol B.* 1976;54(3):395–401 10.1016/0305-0491(76)90264-9 1277808

[7] Arita M Yoshida M Hong S: Resolvin E1, an endogenous lipid mediator derived from omega-3 eicosapentaenoic acid, protects against 2,4,6-trinitrobenzene sulfonic acid-induced colitis. *Proc Natl Acad Sci U S A.* 2005;102(21):7671–7676 10.1073/pnas.0409271102 15890784 PMC1103706

[8] Aktas H Halperin JA: Translational regulation of gene expression by omega-3 fatty acids. *J Nutr.* 2004;134(9):2487S–2491S 15333747 10.1093/jn/134.9.2487S

[9] Tsuji M Murota SI Morita I: Docosapentaenoic acid (22:5, n-3) suppressed tube-forming activity in endothelial cells induced by vascular endothelial growth factor. *Prostaglandins Leukot Essent Fatty Acids.* 2003;68(5):337–342 10.1016/S0952-3278(03)00025-5 12711251

[10] Kitajka K Puskas LG Zvara A: The role of n-3 polyunsaturated fatty acids in brain: modulation of rat brain gene expression by dietary n-3 fatty acids. *Proc Natl Acad Sci U S A.* 2002;99(5):2619–2624 10.1073/pnas.042698699 11880617 PMC122397

[11] Fujikawa M Yamazaki K Hamazaki T: Effect of eicosapentaenoic acid ethyl ester on albuminuria in streptozotocin-induced diabetic rats. *J Nutr Sci Vitaminol (Tokyo).* 1994;40(1):49–61 10.1007/BF02544581 8089771

[12] Shimizu H Ohtani K Tanaka Y: Long-term effect of eicosapentaenoic acid ethyl (EPA-E) on albuminuria of non-insulin dependent diabetic patients. *Diabetes Res Clin Pract.* 1995;28(1):35–40 10.1016/0168-8227(95)01056-J 7587910

[13] Koletzko B Mrotzek M Bremer HJ: Fatty acid composition of mature human milk in Germany. *Am J Clin Nutr.* 1988;47(6):954–959 3376910 10.1093/ajcn/47.6.954

[14] Davidson MH: Omega-3 fatty acids: new insights into the pharmacology and biology of docosahexaenoic acid, docosapentaenoic acid, and eicosapentaenoic acid. *Curr opin lipidol.* 2013;24(6):467–474 10.1097/MOL.0000000000000019 24184945

[15] Kaur G Sinclair AJ Cameron-Smith D: Docosapentaenoic acid (22: 5n-3) down-regulates the expression of genes involved in fat synthesis in liver cells. *Prostaglandins Leukot Essent Fatty Acids.* 2011;85(3–4):155–161 10.1016/j.plefa.2011.06.002 21807486

[16] Lovegrove JA Griffin BA: The acute and long-term effects of dietary fatty acids on vascular function in health and disease. *Curr Opin Clin Nutr Metab Care.* 2013;16(2):162–167 10.1097/MCO.0b013e32835c5f29 23299700

[17] Nicholson T Khademi H Moghadasian MH: The role of marine n-3 fatty acids in improving cardiovascular health: a review. *Food Funct.* 2013;4(3):357–365 10.1039/c2fo30235g 23325431

[18] De Caterina R: n-3 fatty acids in cardiovascular disease. *N Engl J Med.* 2011;364(25):2439–2450 10.1056/NEJMra1008153 21696310

[19] Mohrhauer H Holman RT: Tracer Experiments to Assess Metabolic Conversions of Polyunsaturated Fatty Acids. *J Am Oil Chem Soc.* 1965;42:639–643 10.1007/BF02541306 14328363

[20] Horton JD Shah NA Warrington JA: Combined analysis of oligonucleotide microarray data from transgenic and knockout mice identifies direct SREBP target genes. *Proc Natl Acad Sci U S A.* 2003;100(21):12027–12032 10.1073/pnas.1534923100 14512514 PMC218707

[21] Wang Y Botolin D Christian B: Tissue-specific, nutritional, and developmental regulation of rat fatty acid elongases. *J Lipid Res.* 2005;46(4):706–715 10.1194/jlr.M400335-JLR200 15654130 PMC2430181

[22] Voss A Reinhart M Sankarappa S: The metabolism of 7,10,13,16,19-docosapentaenoic acid to 4,7,10,13,16,19-docosahexaenoic acid in rat liver is independent of a 4-desaturase. *J Biol Chem.* 1991;266(30):19995–20000 1834642

[23] Christensen E Woldseth B Hagve TA: Peroxisomal beta-oxidation of polyunsaturated long chain fatty acids in human fibroblasts. The polyunsaturated and the saturated long chain fatty acids are retroconverted by the same acyl-CoA oxidase. *Scand J Clin Lab Invest Suppl.* 1993;215:61–74 8327852 10.3109/00365519309090698

[24] Reddy JK Hashimoto T: Peroxisomal beta-oxidation and peroxisome proliferator-activated receptor alpha: an adaptive metabolic system. *Annu Rev Nutr.* 2001;21:193–230 10.1146/annurev.nutr.21.1.193 11375435

[25] Kaur G Cameron-Smith D Garg M: Docosapentaenoic acid (22: 5n-3): a review of its biological effects. *Prog Lipid Res.* 2011;50(1):28–34 10.1016/j.plipres.2010.07.004 20655949

[26] Augustsson K Michaud DS Rimm EB: A prospective study of intake of fish and marine fatty acids and prostate cancer. *Cancer Epidemiol Biomarkers Prev.* 2003;12(1):64–67 12540506

[27] de Deckere EA: Possible beneficial effect of fish and fish n-3 polyunsaturated fatty acids in breast and colorectal cancer. *Eur J Cancer Prev.* 1999;8(3):213–221 10.1097/00008469-199906000-00009 10443950

[28] Caygill CP Hill MJ: Fish, n-3 fatty acids and human colorectal and breast cancer mortality. *Eur J Cancer Prev.* 1995;4(4):329–332 10.1097/00008469-199508000-00008 7549825

[29] Berquin IM Min Y Wu R: Modulation of prostate cancer genetic risk by omega-3 and omega-6 fatty acids. *J Clin Invest.* 2007;117(7):1866–1875 10.1172/JCI31494 17607361 PMC1890998

[30] Pala V Krogh V Muti P: Erythrocyte membrane fatty acids and subsequent breast cancer: a prospective Italian study. *J Natl Cancer Inst.* 2001;93(14):1088–1095 10.1093/jnci/93.14.1088 11459870

[31] Colomer R Moreno-Nogueira JM García-Luna PP: N-3 fatty acids, cancer and cachexia: a systematic review of the literature. *Br J Nutr.* 2007;97(5):823–831 10.1017/S000711450765795X 17408522

[32] Ryan AM Reynolds JV Healy L: Enteral nutrition enriched with eicosapentaenoic acid (EPA) preserves lean body mass following esophageal cancer surgery: results of a double-blinded randomized controlled trial. *Ann Surg.* 2009;249(3):355–363 10.1097/SLA.0b013e31819a4789 19247018

[33] Morin C Rousseau E Fortin S: Anti-proliferative effects of a new docosapentaenoic acid monoacylglyceride in colorectal carcinoma cells. *Prostaglandins Leukot Essent Fatty Acids.* 2013;89(4):203–213 10.1016/j.plefa.2013.07.004 23932824

[34] Dietary supplementation with n-3 polyunsaturated fatty acids and vitamin E after myocardial infarction: results of the GISSI-Prevenzione trial. Gruppo Italiano per lo Studio della Sopravvivenza nell’Infarto miocardico. *Lancet.* 1999;354(9177):447–455 10.1016/S0140-6736(99)07072-5 10465168

[35] Marchioli R Barzi F Bomba E: Early protection against sudden death by n-3 polyunsaturated fatty acids after myocardial infarction: time-course analysis of the results of the Gruppo Italiano per lo Studio della Sopravvivenza nell’Infarto Miocardico (GISSI)-Prevenzione. *Circulation.* 2002;105(16):1897–1903 10.1161/01.CIR.0000014682.14181.F2 11997274

[36] Appel LJ Miller ER 3rd Seidler AJ: Does supplementation of diet with ‘fish oil’ reduce blood pressure? A meta-analysis of controlled clinical trials. *Arch Intern Med.* 1993;153(12):1429–1438 10.1001/archinte.1993.00410120017003 8141868

[37] Oda E Hatada K Katoh K: A case-control pilot study on n-3 polyunsaturated fatty acid as a negative risk factor for myocardial infarction. *Int Heart J.* 2005;46(4):583–591 10.1536/ihj.46.583 16157949

[38] Rissanen T Voutilainen S Nyyssonen K: Fish oil-derived fatty acids, docosahexaenoic acid and docosapentaenoic acid, and the risk of acute coronary events: the Kuopio ischaemic heart disease risk factor study. *Circulation.* 2000;102(22):2677–2679 10.1161/01.CIR.102.22.2677 11094031

[39] Oda E Hatada K Kimura J: Relationships between serum unsaturated fatty acids and coronary risk factors: negative relations between nervonic acid and obesity-related risk factors. *Int Heart J.* 2005;46(6):975–985 10.1536/ihj.46.975 16394593

[40] Simon JA Hodgkins ML Browner WS: Serum fatty acids and the risk of coronary heart disease. *Am J Epidemiol.* 1995;142(5):469–476 7677125 10.1093/oxfordjournals.aje.a117662

[41] Sun Q Ma J Campos H: Blood concentrations of individual long-chain n-3 fatty acids and risk of nonfatal myocardial infarction. *Am J Clin Nutr.* 2008;88(1):216–223 18614744 10.1093/ajcn/88.1.216 PMC6598701

[42] Paganelli F Maixent JM Duran MJ: Altered erythrocyte n-3 fatty acids in Mediterranean patients with coronary artery disease. *Int J Cardiol.* 2001;78(1):27–32 10.1016/S0167-5273(00)00442-3 11259810

[43] Mozaffarian D Lemaitre RN King IB: Circulating long-chain omega-3 fatty acids and incidence of congestive heart failure in older adults: the cardiovascular health study: a cohort study. *Ann Intern Med.* 2011;155(3):160–170 10.7326/0003-4819-155-3-201108020-00006 21810709 PMC3371768

[44] Amano T Matsubara T Uetani T: Impact of omega-3 polyunsaturated fatty acids on coronary plaque instability: an integrated backscatter intravascular ultrasound study. *Atherosclerosis.* 2011;218(1):110–116 10.1016/j.atherosclerosis.2011.05.030 21684546

[45] Leng GC Horrobin DF Fowkes FG: Plasma essential fatty acids, cigarette smoking, and dietary antioxidants in peripheral arterial disease. A population-based case-control study. *Arterioscler Thromb.* 1994;14(3):471–478 10.1161/01.ATV.14.3.471 8123654

[46] Hino A Adachi H Toyomasu K: Very long chain N-3 fatty acids intake and carotid atherosclerosis: an epidemiological study evaluated by ultrasonography. *Atherosclerosis.* 2004;176(1):145–149 10.1016/j.atherosclerosis.2004.04.020 15306187

[47] Jackson SP: The growing complexity of platelet aggregation. *Blood.* 2007;109(12):5087–5095 10.1182/blood-2006-12-027698 17311994

[48] Akiba S Murata T Kitatani K: Involvement of lipoxygenase pathway in docosapentaenoic acid-induced inhibition of platelet aggregation. *Biol Pharm Bull.* 2000;23(11):1293–1297 11085354 10.1248/bpb.23.1293

[49] Phang M Garg ML Sinclair AJ: Inhibition of platelet aggregation by omega-3 polyunsaturated fatty acids is gender specific-Redefining platelet response to fish oils. *Prostaglandins Leukot Essent Fatty Acids.* 2009;81(1):35–40 10.1016/j.plefa.2009.05.001 19481915

[50] Lamalice L Le Boeuf F Huot J: Endothelial cell migration during angiogenesis. *Circ Res.* 2007;100(6):782–794 10.1161/01.RES.0000259593.07661.1e 17395884

[51] Kanayasu-Toyoda T Morita I Murota S: Docosapentaenoic acid (22:5, n-3), an elongation metabolite of eicosapentaenoic acid (20:5, n-3), is a potent stimulator of endothelial cell migration on pretreatment *in vitro*. *Prostaglandins Leukot Essent Fatty Acids.* 1996;54(5):319–325 10.1016/S0952-3278(96)90045-9 8832760

[52] Gottlicher M Widmark E Li Q: Fatty acids activate a chimera of the clofibric acid-activated receptor and the glucocorticoid receptor. *Proc Natl Acad Sci U S A.* 1992;89(10):4653–4657 10.1073/pnas.89.10.4653 1316614 PMC49141

[53] Horton JD Goldstein JL Brown MS: SREBPs: transcriptional mediators of lipid homeostasis. *Cold Spring Harb Symp Quant Biol.* 2002;67:491–498 10.1101/sqb.2002.67.491 12858575

[54] Pawar A Jump DB: Unsaturated fatty acid regulation of peroxisome proliferator-activated receptor alpha activity in rat primary hepatocytes. *J Biol Chem.* 2003;278(38):35931–35939 10.1074/jbc.M306238200 12853447

[55] Chen JN Jiang Y Liang Y: DPA n-3, DPA n-6 and DHA improve lipoprotein profiles and aortic function in hamsters fed a high cholesterol diet. *Atherosclerosis.* 2012;221(2):397–404 10.1016/j.atherosclerosis.2012.01.005 22284366

[56] Kishida E Tajiri M Masuzawa Y: Docosahexaenoic acid enrichment can reduce L929 cell necrosis induced by tumor necrosis factor. *Biochim Biophys Acta.* 2006;1761(4):454–462 10.1016/j.bbalip.2006.03.023 16698313

[57] Damsgaard CT Lauritzen L Kjaer TM: Fish oil supplementation modulates immune function in healthy infants. *J Nutr.* 2007;137(4):1031–1036 17374672 10.1093/jn/137.4.1031

[58] Wall R Ross RP Fitzgerald GF: Fatty acids from fish: the anti-inflammatory potential of long-chain omega-3 fatty acids. *Nutr Rev.* 2010;68(5):280–289 10.1111/j.1753-4887.2010.00287.x 20500789

[59] Ruggiero C Lattanzio F Lauretani F: Omega-3 polyunsaturated fatty acids and immune-mediated diseases: inflammatory bowel disease and rheumatoid arthritis. *Curr Pharm Des.* 2009;15(36):4135–4148 10.2174/138161209789909746 20041815

[60] Dangi B Obeng M Nauroth JM: Biogenic synthesis, purification, and chemical characterization of anti-inflammatory resolvins derived from docosapentaenoic acid (DPAn-6). *J Biol Chem.* 2009;284(22):14744–14759 10.1074/jbc.M809014200 19324874 PMC2685656

[61] Amminger GP Schäfer MR Papageorgiou K: Long-chain omega-3 fatty acids for indicated prevention of psychotic disorders: a randomized, placebo-controlled trial. *Arch Gen Psychiatry.* 2010;67(2):146–154 10.1001/archgenpsychiatry.2009.192 20124114

[62] Assies J Lieverse R Vreken P: Significantly reduced docosahexaenoic and docosapentaenoic acid concentrations in erythrocyte membranes from schizophrenic patients compared with a carefully matched control group. *Biol Psychiatry.* 2001;49(6):510–522 10.1016/S0006-3223(00)00986-0 11257236

[63] Lin PY Su KP: A meta-analytic review of double-blind, placebo-controlled trials of antidepressant efficacy of omega-3 fatty acids. *J Clin Psychiatry.* 2007;68(7):1056–1061 10.4088/jcp.v68n0712 17685742

[64] Taepavarapruk P Song C: Reductions of acetylcholine release and nerve growth factor expression are correlated with memory impairment induced by interleukin-1beta administrations: effects of omega-3 fatty acid EPA treatment. *J Neurochem.* 2010;112(4):1054–1064 10.1111/j.1471-4159.2009.06524.x 19968753

[65] Rosenzweig ES Barnes CA: Impact of aging on hippocampal function: plasticity, network dynamics, and cognition. *Prog Neurobiol.* 2003;69(3):143–179 10.1016/S0301-0082(02)00126-0 12758108

[66] Kelly L Grehan B Chiesa AD: The polyunsaturated fatty acids, EPA and DPA exert a protective effect in the hippocampus of the aged rat. *Neurobiol Aging.* 2011;32(12):2318.e1–2318.e15 10.1016/j.neurobiolaging.2010.04.001 20570403

[67] Miller E Kaur G Larsen A: A short-term n-3 DPA supplementation study in humans. *Eur J Nutr.* 2013;52(3):895–904 10.1007/s00394-012-0396-3 22729967

