# Supplementary material for: A review of the biologic and pharmacologic role of docosapentaenoic acid n-3
Source: F1000Res. 2014 Aug 19;2:256. Originally published 2013 Nov 25. [Version 2] doi: 10.12688/f1000research.2-256.v2 (PMC4162505; doi:10.12688/f1000research.2-256.v2)
Supplement: Peer review history

# Peer review: Referee response for version 2

**DOI:** 10.5256/f1000research.5230.r5855

**Published:** 2014-09-08

**Authors:** Philip Calder

**Affiliations:** 1 Faculty of Medicine, University of Southampton, Southampton, UK

The revisions have greatly improved this manuscript which now adequately distinguishes DPA from the other important omega-3 fatty acids and which now provides an accurate, relevant and timely coverage of the possible important functional role of DPA.

I have read this submission. I believe that I have an appropriate level of expertise to confirm that it is of an acceptable scientific standard.

# Peer review: Referee response for version 2

**DOI:** 10.5256/f1000research.5230.r4765

**Published:** 2014-08-20

**Authors:** Bruce Bistrian

**Affiliations:** 1 Nutrition/Infection Laboratory, Department of Medicine, Beth Israel Deaconess Medical Center, Boston, MA, USA

The changes made meet my concerns. This is a useful article.

I have read this submission. I believe that I have an appropriate level of expertise to confirm that it is of an acceptable scientific standard.

# Peer review: Referee response for version 1

**DOI:** 10.5256/f1000research.2985.r4785

**Published:** 2014-05-19

**Authors:** Bruce Bistrian

**Affiliations:** 1 Nutrition/Infection Laboratory, Department of Medicine, Beth Israel Deaconess Medical Center, Boston, MA, USA

In general this article does make a good point that DPA may well be an important omega 3 fatty acid and provides references from in vitro studies, animal studies, and some human epidemiologic studies that DPA may have some unique, beneficial properties. However many of the studies quoted for support in cardiovascular disease confuse association with causation, and this should be noted. Moreover there is still some controversy about the extent of the effectiveness of very long chain omega 3 fatty acids in the improvement of cardiovascular health according to different health authorities. That said the article does provide a useful review that should promote more research into this potentially important fatty acid. As an additional comment there are some misstatements which are presumably typographic on the first page. The retroconversion is DPA to EPA rather than the reverse. Also there is only one elongase necessary to convert EPA to DHA.

I have read this submission. I believe that I have an appropriate level of expertise to confirm that it is of an acceptable scientific standard, however I have significant reservations, as outlined above.

# Peer review: Referee response for version 1

**DOI:** 10.5256/f1000research.2985.r3468

**Published:** 2014-02-17

**Authors:** Philip Calder

**Affiliations:** 1 Faculty of Medicine, University of Southampton, Southampton, UK

There remains significant interest in the health impact of omega-3 fatty acids from oily fish and fish oil type supplements on human health. The physiologic and health benefits are usually ascribed to EPA and DHA. However a third fatty acid, DPA, is present and this has been relatively little researched. Some new work on DPA has been performed which has suggested that it has some unique functional properties. These are reviewed elsewhere, but new updates, views and insights are always to be welcomed. This article aims to provide a current review of DPA and, I think, to distinguish it from EPA and DHA. To be frank, I do not think it achieves the latter aim as well as it might (see comments below).

Specific comments:

1. Title. If the "biologic" is used then this should be countered by "pharmacologic".
2. Title. Should clarify that this is about DPA n-3 not DPA n-6.
3. Abstract. We are told in line 1 that fish oil contains a complex mixture of EPA, DPA and DHA. Therefore it cannot be correct to state on line 5 that ‘It is not correct to state that work with fish oil " *was previously done on EPA and DHA*". Perhaps the author means that the work has focussed on EPA and DHA as the active components?     
4. The last sentence of the abstract makes the same error. We are told first (line 1) that fish oil contains EPA, DPA and DHA. Thus it cannot be correct to state that traditional supplementation is " *with DHA and EPA*" - it is with all three marine omega-3 fatty acids. Perhaps the author means that DPA has "separate and distinct properties compared with EPA and DHA"? 
5. Page 2, column 1, 5 and 2 lines from the bottom. ‘b’ in b-oxidation should be shown as the Greek symbol beta.
6. Page 2, column 1, 3 lines from bottom. I think " *DPA from EPA*" should read "DPA from DHA" since the former is not "reverse" metabolism and would not involve beta-oxidation.
7. Page 2, column 2, line 3. What is meant by " *Clinical associations*"? Does the author mean "Clinical effects"?
8. The author is setting out to establish that DPA has independent functional features. However in many parts the evidence for this is based upon rehearsing the effects of fish oil supplements - these data cannot be used to say DPA has effects, since the effects may be due to any one of EPA, DPA and DHA or indeed to all three. Too often effects are due to "n-3 fatty acids" and it is then concluded that DPA is functional. I would much rather that the author is explicit in picking out and describing those studies where DPA is shown to have a function. A really poor example is in the first paragraph about cardiovascular disease where the effects seen in the GISSI trial are described. It is NOT acknowledged that these can have nothing to do with DPA since the preparation used in that study was a pharmaceutical preparation composed almost entirely of ethyl esters of EPA and DHA. To say otherwise is to mislead readers. 
9. Page 4, column 1, line 31. Should "en" read "ten"?
10. Page 4, column 1, 4 lines from bottom. Please add "in hamsters" after "50%".

I have read this submission. I believe that I have an appropriate level of expertise to confirm that it is of an acceptable scientific standard, however I have significant reservations, as outlined above.
